# SSBER: removing batch effect for single-cell RNA sequencing data

**DOI:** 10.1186/s12859-021-04165-w

**Published:** 2021-05-14

**Authors:** Yin Zhang, Fei Wang

**Affiliations:** 1 Shanghai Key Lab of Intelligent Information Processing, Shanghai, China; 2 School of Computer Science and Technology, Fudan University, Shanghai, China

**Keywords:** Data integration, Batch effect, The shared cell type, Supervised cell type assignment

## Abstract

**Background:**

With the continuous maturity of sequencing technology, different laboratories or different sequencing platforms have generated a large amount of single-cell transcriptome sequencing data for the same or different tissues. Due to batch effects and high dimensions of scRNA data, downstream analysis often faces challenges. Although a number of algorithms and tools have been proposed for removing batch effects, the current mainstream algorithms have faced the problem of data overcorrection when the cell type composition varies greatly between batches.

**Results:**

In this paper, we propose a novel method named SSBER by utilizing biological prior knowledge to guide the correction, aiming to solve the problem of poor batch-effect correction when the cell type composition differs greatly between batches.

**Conclusions:**

SSBER effectively solves the above problems and outperforms other algorithms when the cell type structure among batches or distribution of cell population varies considerably, or some similar cell types exist across batches.

## Background

In 2009, Tang et al. developed the first sequencing technology for single-cell RNA sequencing (scRNA-seq). Unlike traditional "bulk" RNA sequencing in the past, scRNA-seq measures the expression of each gene from the perspective of a single cell [1]. With the rapid development of biotechnology, single-cell RNA sequencing (scRNA-seq) has become one of the most prioritized sequencing research directions in recent years [2, 3]. It is meaningful to analysis scRNA-seq data, facilitating to understand the biological heterogeneity and to discover new cell types. In the process of data analysis, integrating multiple batches can contain more biological information, which will help us to obtain more reliable downstream analysis results. However, biological data can be easily affected by systematic variations especially due to experimental technology deviations or artificial errors [4]. Effectively removing batch effects can reduce the influence of technical or artificial errors in the process of analyzing scRNA-seq data [5].

Traditional methods to remove batch effect for bulk RNA-seq data are not a good option for scRNA-seq data since data characteristics are distinct, for example, scRNA-seq data is very sparse with a complex distribution. Some approaches especial for scRNA-seq data have been developed, including anchor-based methods, clustering-based methods and deep learning methods. Anchor-based methods identify anchors between batches, which are two cells from different batch and these two cells are mutual nearest neighbors. The batch effect is represented by the difference vectors of anchor pairs. Representative algorithms of this category include MNN [6], Seurat [7, 8], Scanorama [9], BBKNN [10], etc., among which MNN is the first method to adopt this idea. It computes the difference vectors between the anchor pairs identified by K nearest neighbors (KNN) algorithm and uses the Gaussian mixture model to calculate the final corrected vector. Seurat introduces the canonical correlation analysis (CCA) algorithm [11] on the basis of MNN to reduce the raw data to the low-dimensional most relevant subspace, identifies anchor pairs in the subspace and applies the graph weighting algorithm to calculate the final corrected vector. BBKNN presents a random projection tree algorithm that replaces the KNN algorithm to make speed-up. Scanorama uses singular value decomposition (SVD) for dimensionality reduction, identifies anchor pairs in the low dimensionality space, mixes all batches together without the restriction that there is at least one shared cell type in all batches. Harmony [12] and LIGER [13] are clustering-based method. Harmony uses an iterative clustering method and ensures that cells in each cluster come from as many batches as possible during each iteration. LIGER uses a non-negative matrix factorization to maximize shared information between batches and then employs a clustering algorithm to group the shared parts. Most deep learning methods [14, 15] are based on autoencoder or variational autoencoder. They are representative learning which removes noise by data compression and reconstruction.

The methods mentioned above perform well when the batch effect is much smaller than biological variation, since in anchor-based methods, anchors actually can be considered as the  same cell type between batches. If the batches are highly heterogeneous, these methods cannot achieve a satisfactory integration result, since wrong anchors could be popped out based on mutual nearest neighbors and consequently mislead batch correction. And Harmony maximizes batch diversity while some cell types may not be included in a batch. It has been verified in a recent comprehensive analysis and comparison [16, 17] in which the above methods are evaluated through 10 datasets.

**Fig. 1:**
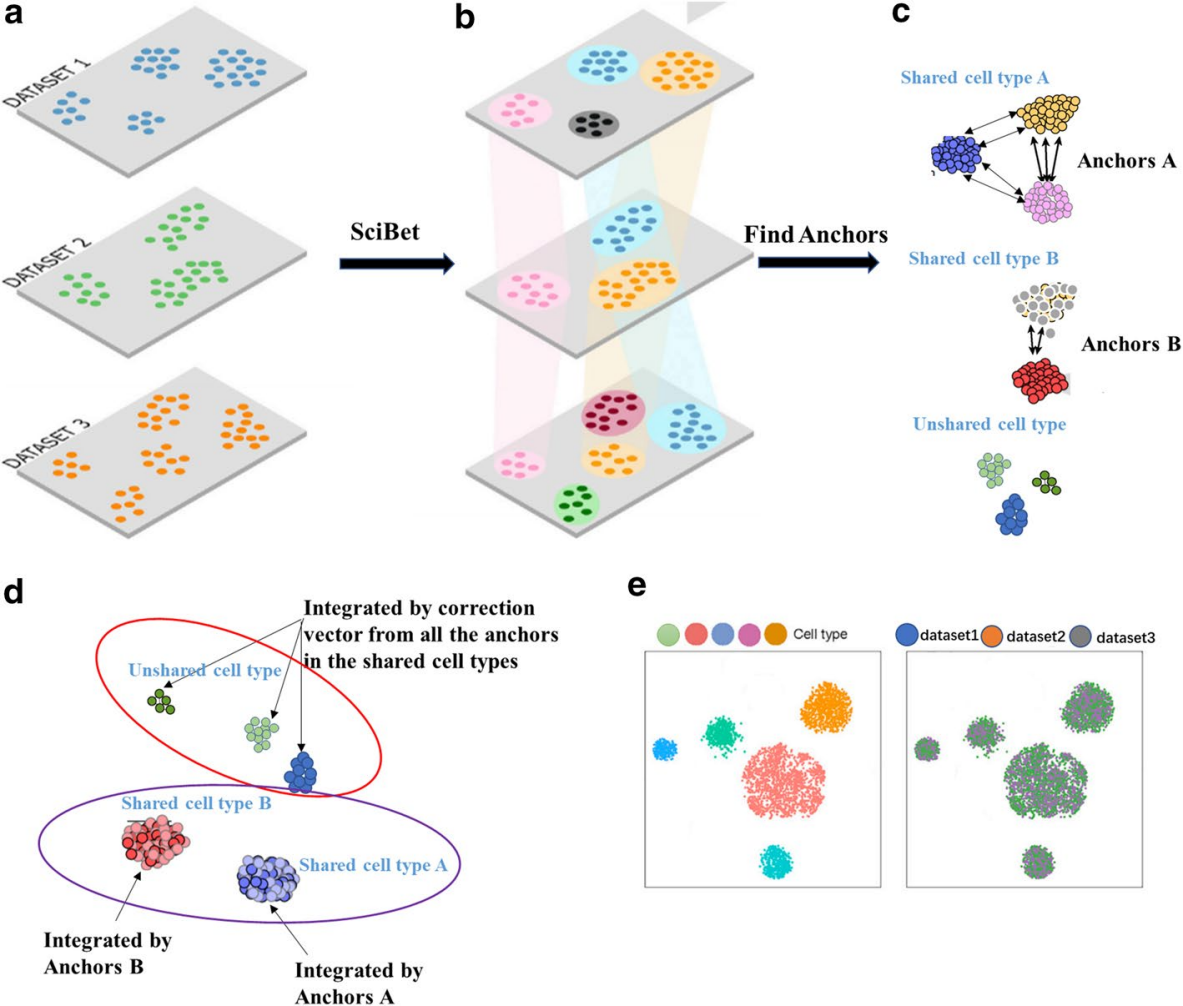
Overview of SSBER**.** **a** Input of the raw data, **b** projecting cell type information via SciBet, **c** detecting anchor pairs—based on the distance of cells from shared cell type, **d** integrating data guided by correction vectors from anchor pairs, **e** plots of integrated data

With the emergence of single cell atlas, tsunamic data with cell type label definitely could provide prior information for new data integration and bring biological interpretability. In this paper, a new algorithm named SSBER, that introduces biological priori information, for single cell RNA-seq dataset integration is proposed, aiming to improve batch-effect correction when high heterogeneity exist among batches (The overall process of SSBER is shown in Fig. 1). Experiments on various datasets in different scenarios show that: (1) when the cell type composition differs greatly among batches, SSBER performs better than other algorithms, such as Harmony, Seurat and LIGER. (2) When similar cell types exist among batches or quantity distributions of cells from various cell types are seriously unbalanced, SSBER also outperforms other algorithms.

## Results

To give a comprehension evaluation of SSBER, we implement some experiments on real data under three scenarios, cell-type structure across batches is not identical, similar cell types across batches exists, and quantity distribution of cells from various cell types is seriously unbalanced. And we apply SSBER to time-series datasets for comparing the variation of development trajectory, in particular compared to Harmony.

### Evaluation metrics

Evaluation metrics are composed of two categories, removal of batch effects and conversation of biological variance [18]. The first category includes the k-nearest neighbor batch-effect test (KBET) [16, 19], local inverse Simpson’s index (LISI) [12, 16], average silhouette width (ASW) [20]. The second category includes adjusted rand index (ARI) [21], isolated label scores, cell cycle variance conservation, and overlaps of highly variable genes (HVGs) per batch before and after integration [18]. Besides t-Distributed Stochastic Neighbor Embedding (t-SNE) [22] as well as Uniform Manifold Approximation and Projection (UMAP) [23] are employed to give visualizations.

1. *K-nearest-neighbor batch estimation (KBET)* KBET measures whether batch mixing is uniform through comparison of local batch label distribution against global batch label distribution. The lower fraction of null hypothesis rejections (range from 0 to 1) represents that the local distribution is more similar to the global distribution, which means better batch mixing around a cell. Following the KBET paper [19], we respectively choose 5%, 10%, 15%, 20%, and 25% of the sample size and get the median of all KBET rejection rates to produce the final KBET result for each method.
2. *Local inverse Simpon’s index (LISI)* LISI can be used to assess goodness of batch integration(iLISI) and cell type integration (cLISI) [12, 16]. In the case of iLISI to measure batch mixing, the index is computed for batch labels, and a score closer to the expected number of batches denotes better batch mixing. For cell type LISI (cLISI), the index is computed for all cell type labels, and a score closer to 1 denotes that the clusters contain purer cell types. Code to compute LISI is available at https://github.com/immunogenomics/LISI. We computed the iLISI and cLISI scores for each cell in the dataset, and then determined the median values.
3. *Average silhouette width (ASW)* The ASW indicator is similar to the LISI indicator, which can be used to assess goodness of both batch integration (ASW_batch) and cell type integration (ASW_celltype). The difference between the ASW and LISI indicators is that ASW uses the distance difference between cells within a same cluster and different clusters to measure the distribution of cells. The resulting score ranges from − 1 to 1, where a high score denotes that the cell fits well in the current cluster, while a low score denotes a poor fit. The average score of all data points is used to measure overall cell type purity (ASW_celltype) or batch mixing (ASW_batch) through the choice of labels. In terms of ASW_celltype, the higher score represents the higher purity of the cell type, as for ASW_batch, the lower score denotes a better batch-mixing performance.
4. *Adjusted rand index (ARI)* The ARI is used to evaluate batch correction methods in terms of cell type purity. The score is calculated by using the true cell type label and the predicted cell type label. The higher ARI value denotes higher purity of the cell type.
5. *Isolated label scores* To estimate rare cell identity annotation, isolated label scores evaluate how well the data integration methods dealt with cell identity labels shared by few batches. Specifically, we identified isolated cell labels as the labels present in the least number of batches in the integration task. The score evaluates how well these isolated labels separate from other cell identities. We implemented two versions of the isolated label metric: the isolated label F1 and isolated label ASW, the mean score of two isolated labels is returned as the final score. For specific calculation details, please see the paper [18].
6. *HVG conservation* The highly variable gene (HVG) conservation score is a proxy for the preservation of the biological signal. As in paper [18], we computed the number of HVGs before and after correction for each batch via Scanpy’s highly_variable_genes function (using flavor = “cell ranger”). If available, we identified 500 HVGs per batch. If fewer than 500 genes were present in the integrated object for a batch, the number of HVGs was set to half the total genes in that batch. The overlap coefficient is defined as:
  1 $${\text{overlap}}\left( {{\text{X}},{\text{Y}}} \right) = {{\left| {X \cap Y} \right|} \mathord{\left/ {\vphantom {{\left| {X \cap Y} \right|} {{\text{min}}\left( {\left| X \right|,\left| Y \right|} \right)}}} \right. \kern-\nulldelimiterspace} {{\text{min}}\left( {\left| X \right|,\left| Y \right|} \right)}}$$
  where X and Y denote the HVGs before and after correction. The overall HVG score is the mean of the per-batch HVG overlap coefficients. Since Harmony and LIGER return data after dimension reduction, it is almost impossible to compute HVG score for them, these scores are omitted in Tables 1, 2, 3, 4, 5 and 6.

**Table 1:** Metrics on the human blood dendritic cell dataset

	*Seurat*	*Harmony*	*SSBER*	*LIGER*
iLISI	**1.5804**	1.4912	1.4125	1.342
cLISI	1.3951	1.1733	**1.1652**	1.4322
ARI	0.707	0.7806	**0.8496**	0.6824
ASW_batch	0.064	0.037	**0.031**	0.059
ASW_celltype	0.186	0.336	**0.395**	0.168
Isolated label	0.387	0.416	**0.623**	0.327
Cell cycle	0.473	**0.636**	0.583	0.450
HVG	0.613		**0.624**	

Bold represents the best indicator among four algorithms
7. *Cell cycle conservation* The cell cycle conservation score evaluates how well the cell cycle effect can be scored before and after integration. We computed cell cycle conservation scores using the same calculation process as in paper [18]. Score closes to 0 indicating lower conservation and 1 indicating complete conservation of the variance explained by cell cycle.

### Scenario 1: cell type structure across batches is not identical

**Fig. 2:**
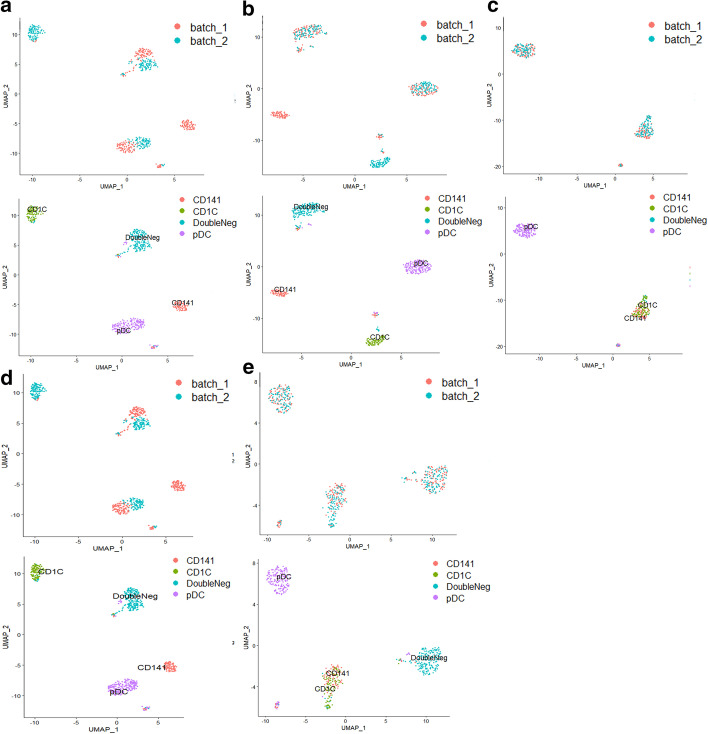
Comparison on the human blood cell dendritic data. **a** Visualization for raw data, **b** UMAP analysis on the integrated data after SSBER, **c** UMAP analysis on the integrated data after Seurat, **d** UMAP analysis on the integrated data after Harmony. **e** UMAP analysis on the integrated data after LIGER

We collected two published datasets, human blood cell dendritic data [24] and human pancreas data [25, 26]. In human blood cell dendritic data, pDC and DoubleNeg are shared cell types in both batches. We delete CD1C cells in the first batch and CD141 cells in the second batch, so they respectively appear in two different batches. As shown in Fig. 2a, the visualization of the raw data, DoubleNeg and pDC are completely separated due to the batch effects. As shown in Fig. 2b, SSBER achieves the best data integration performance. Batch effects are removed, cells of same subpopulation are mixed well and different subpopulations even similar subpopulations are separated. As shown in Fig. 2c, Seurat mixes CD141 and CD1C together after data integration. The main reason is that Seurat mismatches anchors which in return mislead batch correction. As shown in Fig. 2d, although Harmony separates all cell types well while mixing them in batches, some cells of DoubleNeg and pDC are also separated from the main cluster and could be grouped into a new cluster. The reason lies on maximization of batch diversity within a cluster. LIGER mixes CD141 and pDC, CD1C and DoubleNeg together after data integration (Fig. 2e) since it also tries to maximize the shared space across batches.

**Fig. 3:**
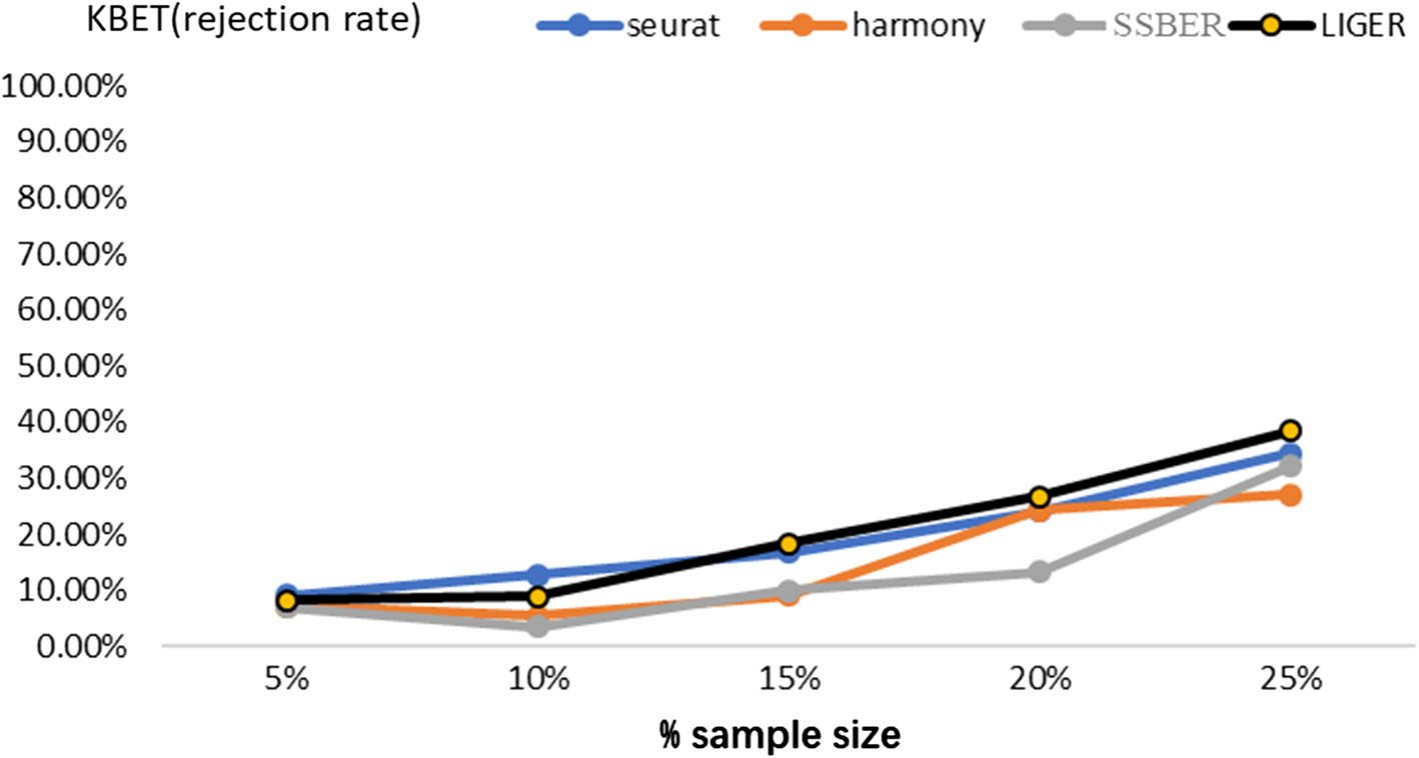
KBET score on the human blood dendritic cell dataset

As shown in Table 1, SSBER achieves the best performance on the cell type purity, cLISI, ASW_celltype and ARI reaching 1.165224, 0.395 and 0.8489714 respectively. Seurat, Harmony, and LIGER mix CD141 and CD1C cells in the integrated data and split pDC cells into two clusters, so the indicators for measuring cell purity are not as good as SSBER. On the metrics indicating batch mixing, SSBER is best on ASW_batch and Seurat is best on iLISI. As for metrics on conversation of biological variance, SSBER achieves the best performance on isolated label score and HVG conservation score, Harmony is the best on cell type conservation score. As for KBET score, shown in Fig. 3, SSBER is basically comparable with Harmony and better than Seurat and LIGER.

In order to further explore the effect of SSBER on the fusion of multiple batches, we reformed human pancreas dataset. Instances of some cell types were removed for each batch, for example, we removed all alpha cells from the celseq dataset, all beta cells from the smartseq2 dataset. After perturbation, acinar is a shared cell type among all batches, delta, beta and ductal cells only appear in two batches, and acitivated_stellate, alpha, endothelial, and gamma belong to only one batch. The detail dataset description is shown in Table 2.

**Table 2:** Detail description on the human pancreas dataset

	*celseq*	*celseq2*	*smartseq2*
Acinar	✓	×	✓
Beta	✓	×	✓
Delta	✓	✓	✓
Activated	✓	✓	×
Alpha	✓	×	×
Ductal	×	✓	✓
Gamma	×	✓	×
Endothelial	×	✓	×

**Fig. 4:**
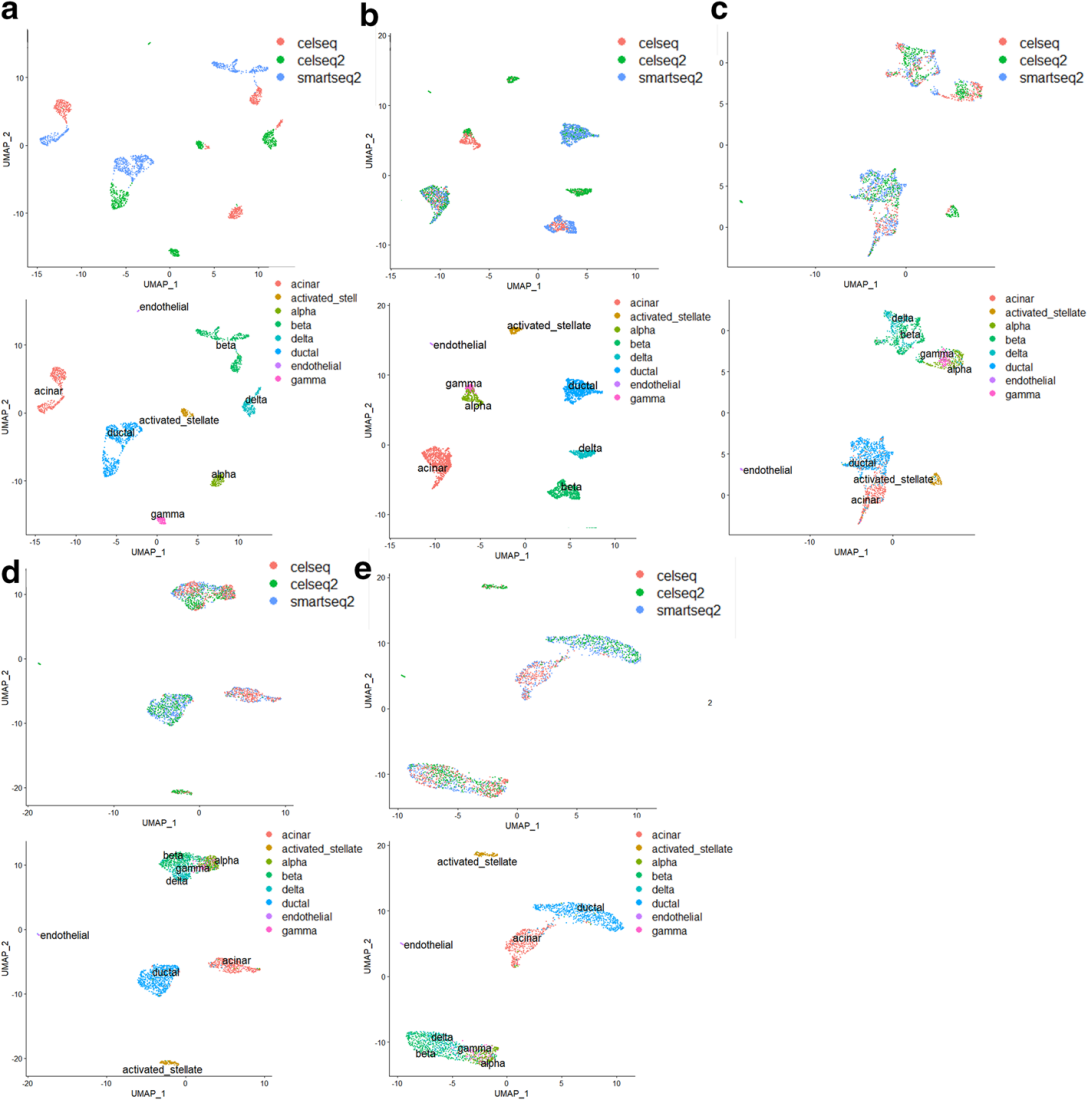
Comparison on the reformed human pancreas dataset. **a** Visualization for raw data, **b** UMAP analysis on the integrated data after SSBER, **c** UAMP analysis on the integrated data after Seurat, **d** UAMP analysis on the integrated data after Harmony. **e** UAMP analysis on the integrated data after LIGER

SSBER, Seurat, Harmony and LIGER were also used to integrate this perturbed dataset. SSBER effectively separates the above cell types with a good batch mixing, shown in Fig. 4b. As shown in Fig. 4c, Seurat mixes alpha with gamma, beta with delta cells. The reason lies on that KNN algorithm pops out some anchor pairs in which two cells are not a same cell type. These wrong anchors lead to the cells from different subpopulations being mixed together. As shown in Fig. 4d, e, Harmony and LIGER totally integrates delta, beta, gamma, and alpha cells into a large cluster. Harmony is a clustering-based algorithm, the objective function of it includes as many batches as possible in a cluster, so when the cell type structure differs greatly among batches, cells from different subpopulations will be mixed incorrectly.

**Fig. 5:**
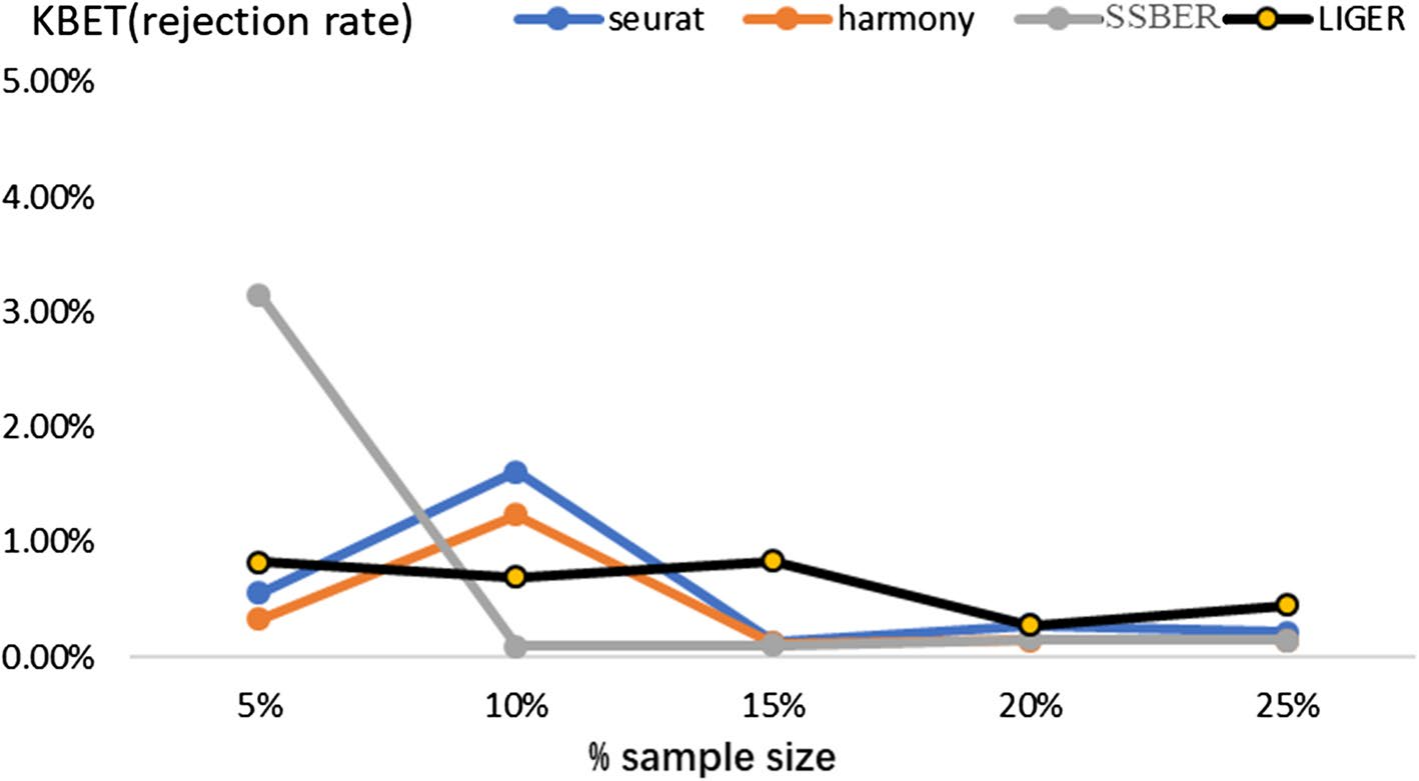
KBET score on the human pancreatic dataset

SSBER is the best one in terms of isolated label, cell type conservation score and HVG conservation score that measure on the conservation of biological variance, shown in Table 3. SSBER is also best in terms of ARI, ASW_celltype and cLISI that measure on the cell type purity. On the metrics indicating the degree of batch mixing, such as ASW_batch and iLISI, superficially SSBER is slightly inferior to Seurat, Harmony and LIGER. The main reason is that when there are many subpopulations that are not shared between batches, SSBER will not mix these cells like other algorithms, but integrate data strictly according to the cell type, while ASW_batch and iLISI only evaluate the uniformity of batch mixing without same cell type constraint, at this time, mixing more cells across batches even wrong mixing that means different subpopulations could reach better score. As for KBET score, shown in Fig. 5, SSBER is basically comparable with Harmony, Seurat and LIGER.

**Table 3:** Metrics on the human pancreas dataset

	*Seurat*	*Harmony*	*SSBER*	*LIGER*
iLISI	**1.878475**	1.864391	1.46051	1.8448
cLISI	1.2320	1.3623	**1.0169**	1.2632
ARI	0.6649	0.5393	**0.8599**	0.5839
ASW_batch	0.026	**0.015**	0.032	0.018
ASW_celltype	0.523	0.404	**0.786**	0.518
Isolated label	0.738	0.702	**0.921**	0.673
Cell cycle	0.606	0.681	**0.703**	0.583
HVG	0.702		**0.747**	

Bold represents the best indicator among four algorithms

It is easy to conclude that, when the cell type structure is heterogeneous among batches, SSBER performs better than Seurat, Harmony and LIGER. If there are unshared subpopulations among batches, iLISI, KBET and ASW_batch scores are not good metrics since they measure the uniformity of batch mixing.

### Scenario 2: similar cell types exist across batches

We collected the human peripheral blood mononuclear dataset [27], in which the cell type structure between batches is basically similar and there are two pairs of similar cell types, CD4 T and CD8 T, Monocyte_CD14 and Monocyte_FCGR3A.

**Fig. 6:**
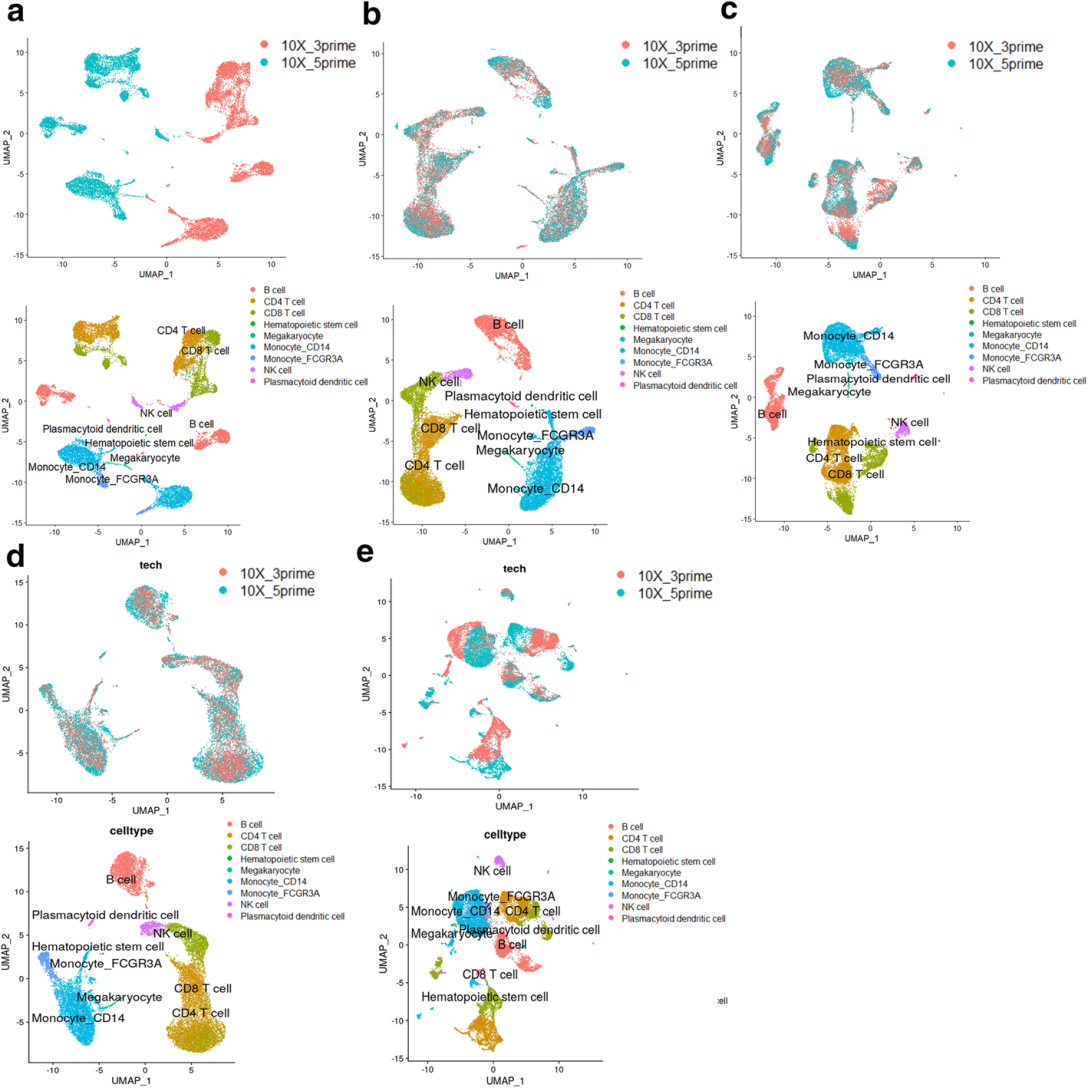
Comparison on the human peripheral blood mononuclear dataset. **a** Visualization for raw data, **b** UMAP analysis on the integrated data after SSBER, **c** UMAP analysis on the integrated data after Seurat, **d** UMAP analysis on the integrated data after Harmony. **e** UMAP analysis on the integrated data after LIGER

As shown in Fig. 6, none of SSBER, Seurat, Harmony and LIGER could generate distinct clusters of Monocyte_CD14 and Monocyte_FCGR3A, or CD4 T and CD8 T in the visualization plots.

**Fig. 7:**
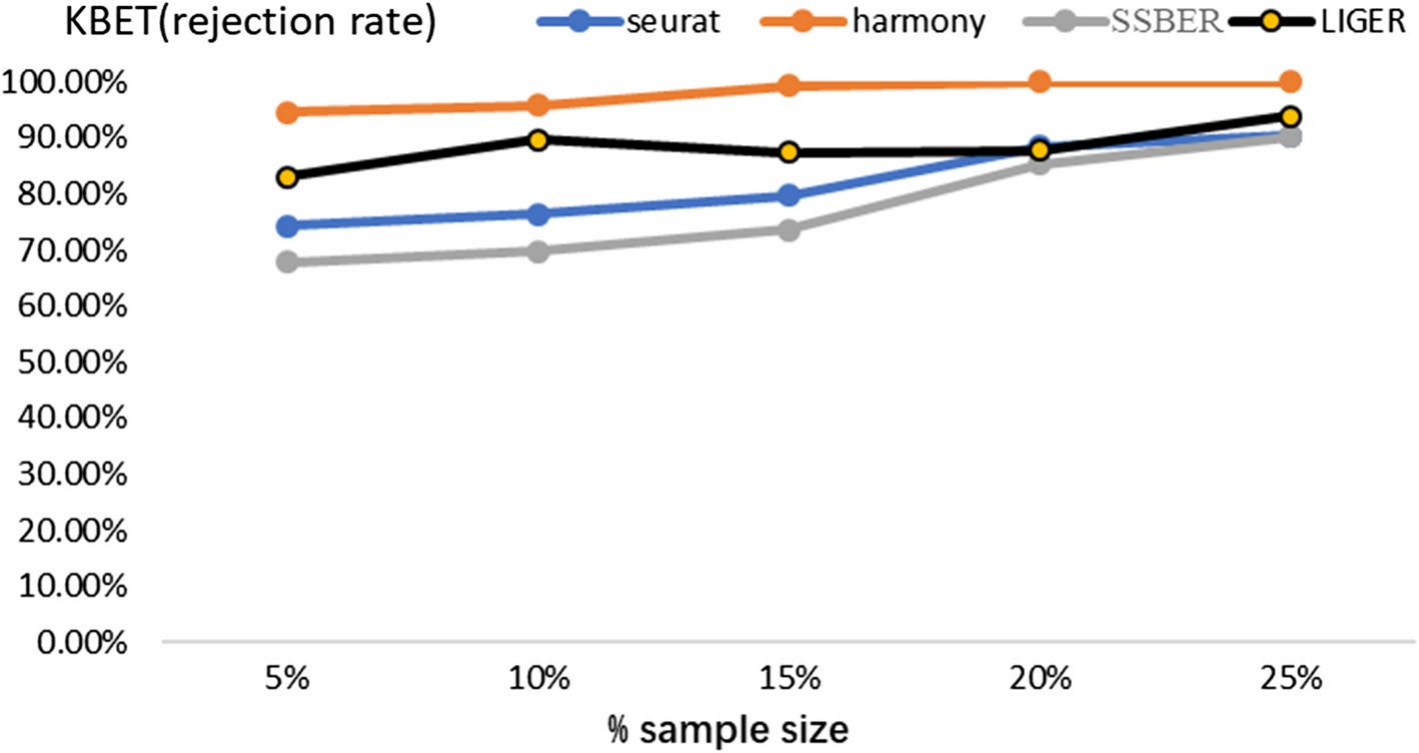
KBET scores on the human peripheral blood mononuclear dataset

As shown in Table 4, SSBER is best, except on iLISI and cell type conservation score. Since CD4 T cells and CD8 T cells are hard to be distinguished, we specially calculated the cLISI score for CD4 T cells and CD8 T cells, Seurat, Harmony, LIGER and SSBER reach 1.1323, 1.2836, 1.224 and 1.377 respectively, SSBER is the best. The KBET score is shown in Fig. 7 and we can see that SSBER is the top method regardless of the sampling ratio, and Seurat ranks the second.

**Table 4:** Metrics on the human peripheral blood mononuclear dataset

	*Seurat*	*Harmony*	*SSBER*	*LIGER*
iLISI	1.458856	1.562768	1.578711	**1.58021**
cLISI	1.074385	1.065975	**1.048792**	1.0723
ARI	0.5720612	0.6247241	**0.696191**	0.61782
ASW_batch	0.064	0.096	**0.056**	0.089
ASW_celltype	0.348	0.329	**0.387**	0.319
Isolated label	0.501	0.583	**0.606**	0.469
Cell cycle	0.674	**0.761**	0.733	0.612
HVG	0.729		**0.815**	

Bold represents the best indicator among four algorithms

### Scenario 3: distribution of cells from various cell types is seriously unbalanced

To compare the data-correction performance of four algorithms when the quantity distribution of cells from various cell types is seriously unbalanced, we collected the mouse retinal cell dataset [16] and the 293t_jurkat cell line dataset [27] as experimental datasets.

**Fig. 8:**
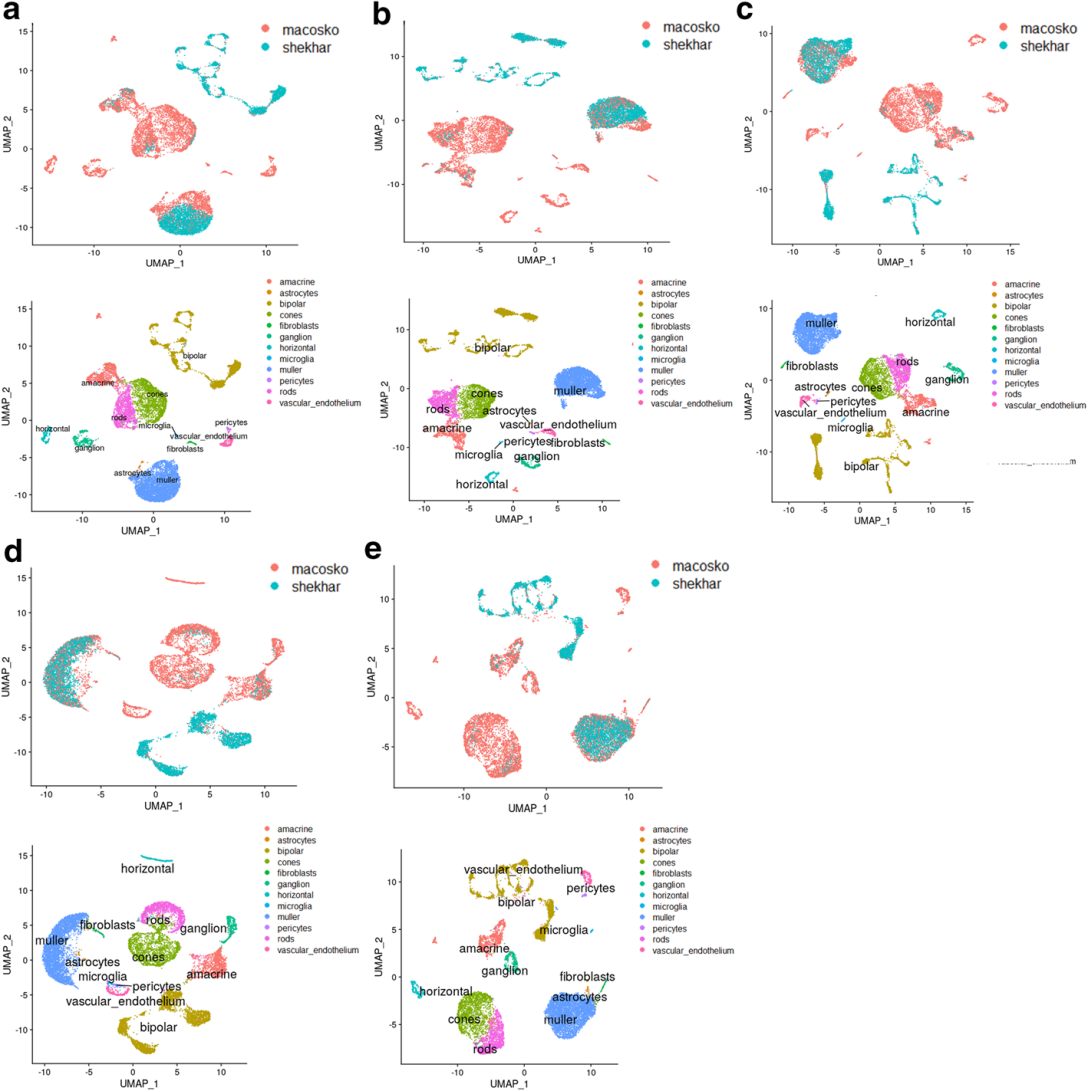
Comparison on the mouse retinal cell dataset. **a** Visualization for raw data, **b** UMAP analysis on the integrated data after SSBER, **c** UMAP analysis on the integrated data after Seurat, **d** UMAP analysis on the integrated data after Harmony. **e** UMAP analysis on the integrated data after LIGER

**Fig. 9:**
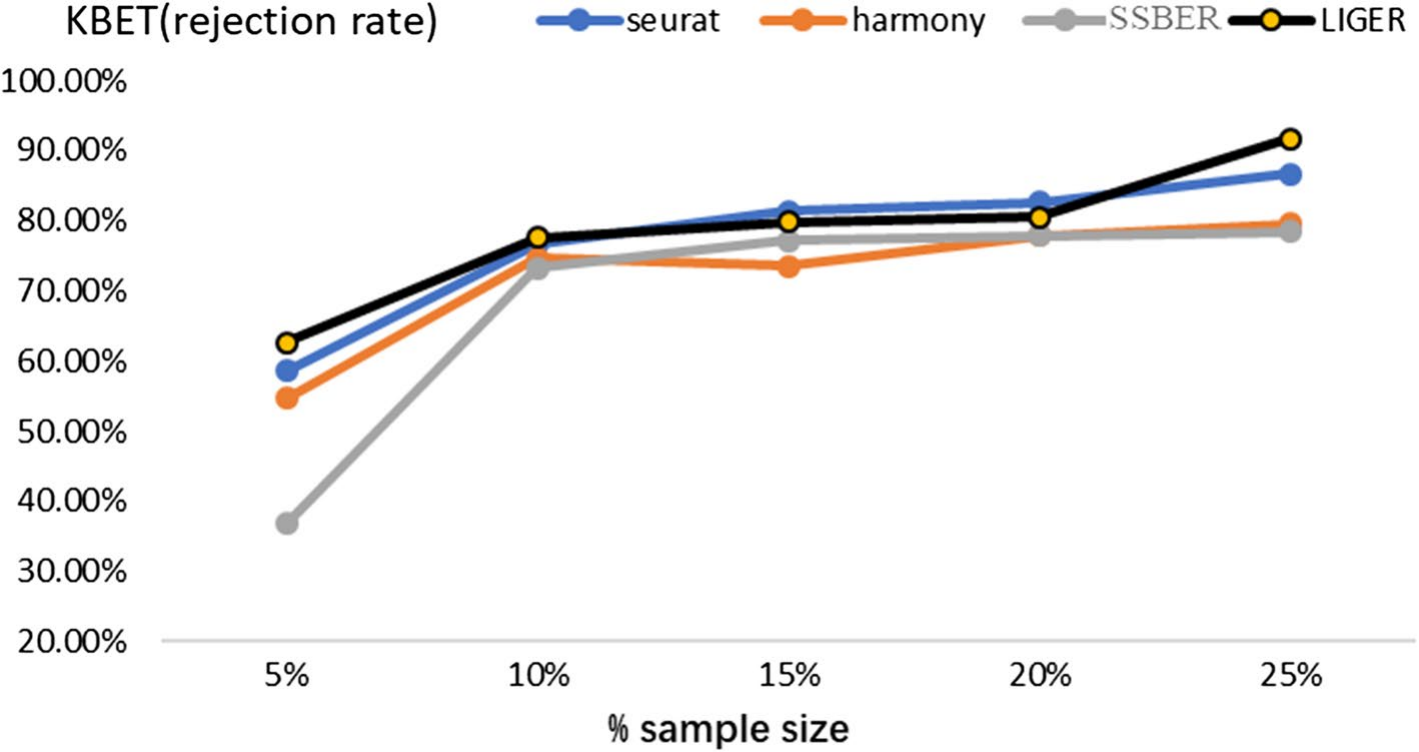
KBET scores on the mouse retina dataset

As shown in Fig. 8, in the mouse retina dataset, bipolar cells monopolizes batch 1 while rod cells monopolizes batch 2, and ganglion, vascular endothelium as well as horizontal cells do not exist in batch 1. Due to the large number of cell types, it is hard to visually distinguish the results after data integration from four algorithms. We count on evaluation metrics. It is shown in Table 5, SSBER outperforms other alorithms on the metrics of cLISI, ARI and ASW_celltype, which reflect the cell type purity. Especially on ARI, SSBER is much better than the other three algorithms. Since some cell types are not shared in both batches, the credibility of iLISI and ASW_batch should be compromised. As for label-free conservation metrics, SSBER achieves the best performance on isolated label score and HVG conservation score, Harmony is the best on cell type conservation score. In the term of KBET, SSBER and Harmony are similar and better than Seurat and LIGER (Fig. 9).

**Table 5:** Metrics on the mouse retina dataset

	*Seurat*	*Harmony*	*SSBER*	*LIGER*
iLISI	1.166076	**1.200744**	1.146624	1.17424
cLISI	1.054624	1.36209	**1.044166**	1.2365
ARI	0.6525991	0.53935	**0.850938**	0.54793
ASW_batch	**0.142**	0.145	0.148	0.184
ASW_celltype	0.673	0.684	**0.765**	0.703
Isolated label	0.592	0.647	**0.783**	0.618
Cell cycle	0.521	**0.587**	0.556	0.538
HVG	0.529		**0.582**	

Bold represents the best indicator among four algorithms

In the 293t_jurkat cell line data, the cell type structure between batches is basically similar and only two cell types 293t and jurkat are contained. The ratio of the number of 293t cells to jurkat cells in batch 1 is 1:9, while this ratio in batch 2 is 5:5.

**Fig. 10:**
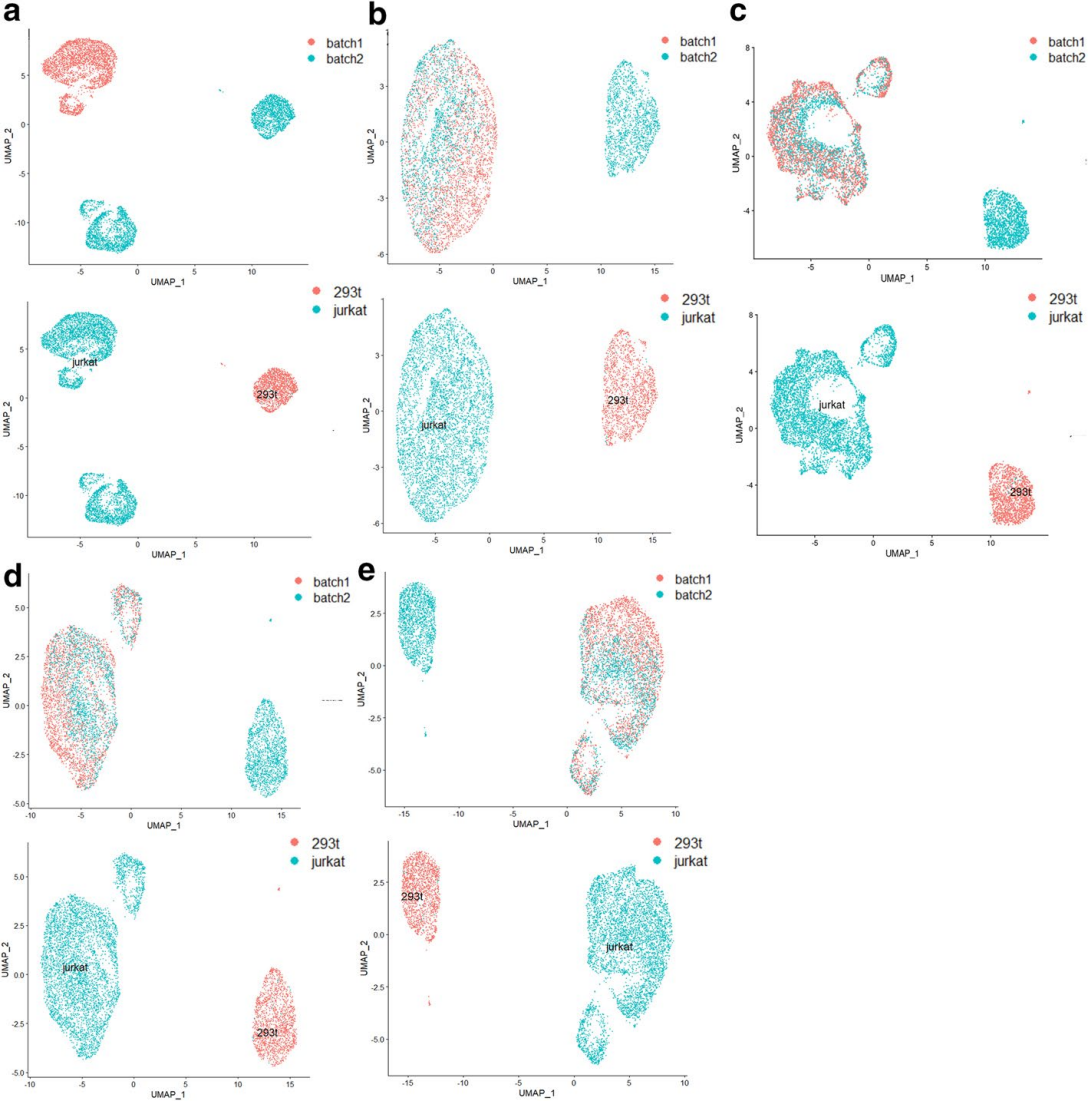
Comparison on the 293t_jurkat cell line data. **a** Visualization for raw data, **b** UMAP analysis on the integrated data after SSBER, **c** UMAP analysis on the integrated data after Seurat, **d** UMAP analysis on the integrated data after Harmony. **e** UMAP analysis on the integrated data after LIGER

**Fig. 11:**
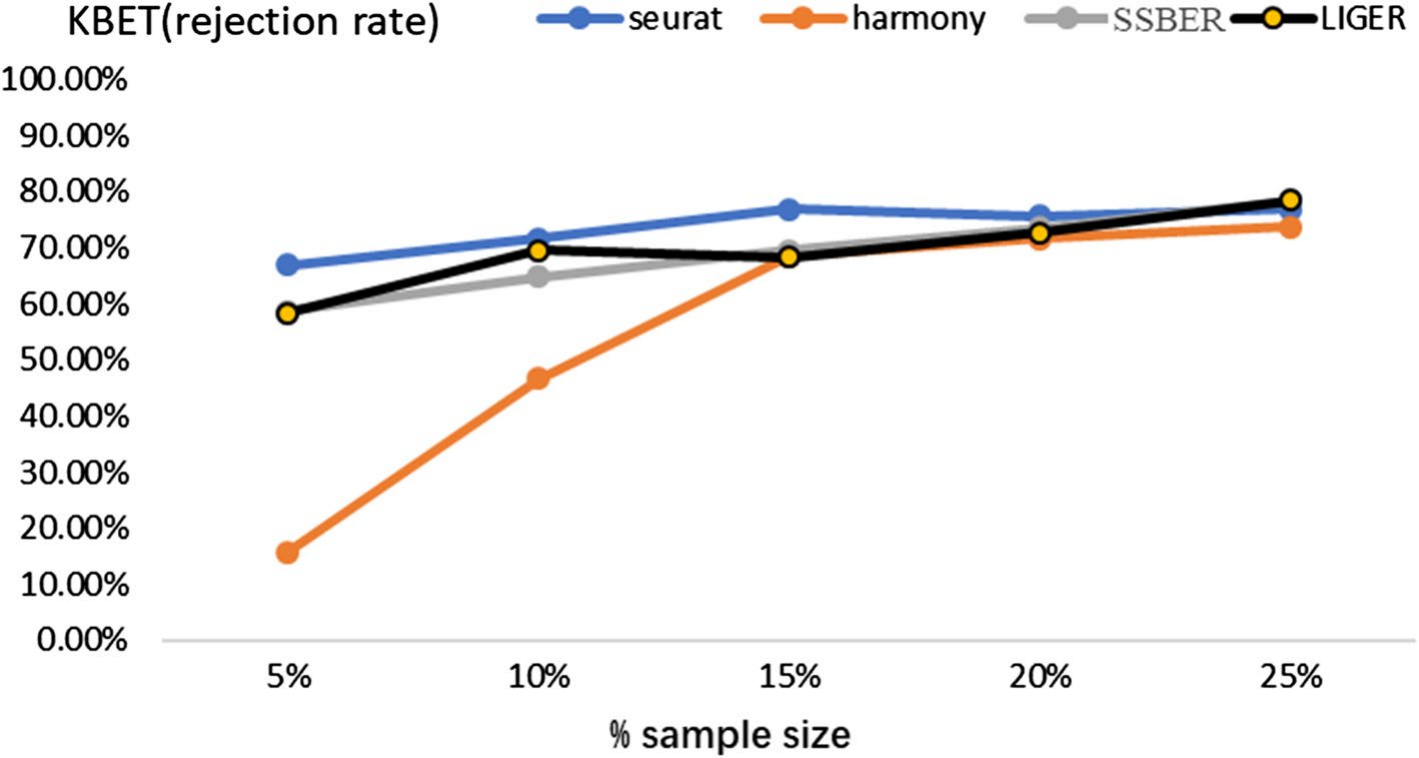
KBET scores on the cell line dataset

It can be seen there are obvious batch effects from the visualization of the raw data (Fig. 10a). Seurat, Harmony and LIGER are more likely to divide jurkat cells into two clusters (Fig. 10c–e), and SSBER gives a much closer group of jurkat cells. It is also shown in Table 6, SSBER gets 0.994 on ARI score, much better than 0.864 of LIGER and 0.885 of Harmony. Besides, on all other metrics, including iLISI, cLISI, ASW_batch, ASW_celltype (Table 6) and KBET (Fig. 11), SSBER is also the best one.

**Table 6:** Metrics on the cell line dataset

	*Seurat*	*Harmony*	*SSBER*	*LIGER*
iLISI	1.369536	1.48654	**1.56786**	1.46342
cLISI	1.005186	1.0045738	**1.000456**	1.00428
ARI	0.7753852	0.885436	**0.993591**	0.86463
ASW_batch	0.167	0.146	**0.086**	0.186
ASW_celltype	0.447	0.668	**0.783**	0.658
Isolated label	0.729	0.858	**0.925**	0.837
Cell cycle	0.618	**0.707**	0.662	0.609
HVG	0.726		**0.784**	

Bold represents the best indicator among four algorithms

### Time course developmental trajectories analysis

**Fig. 12:**
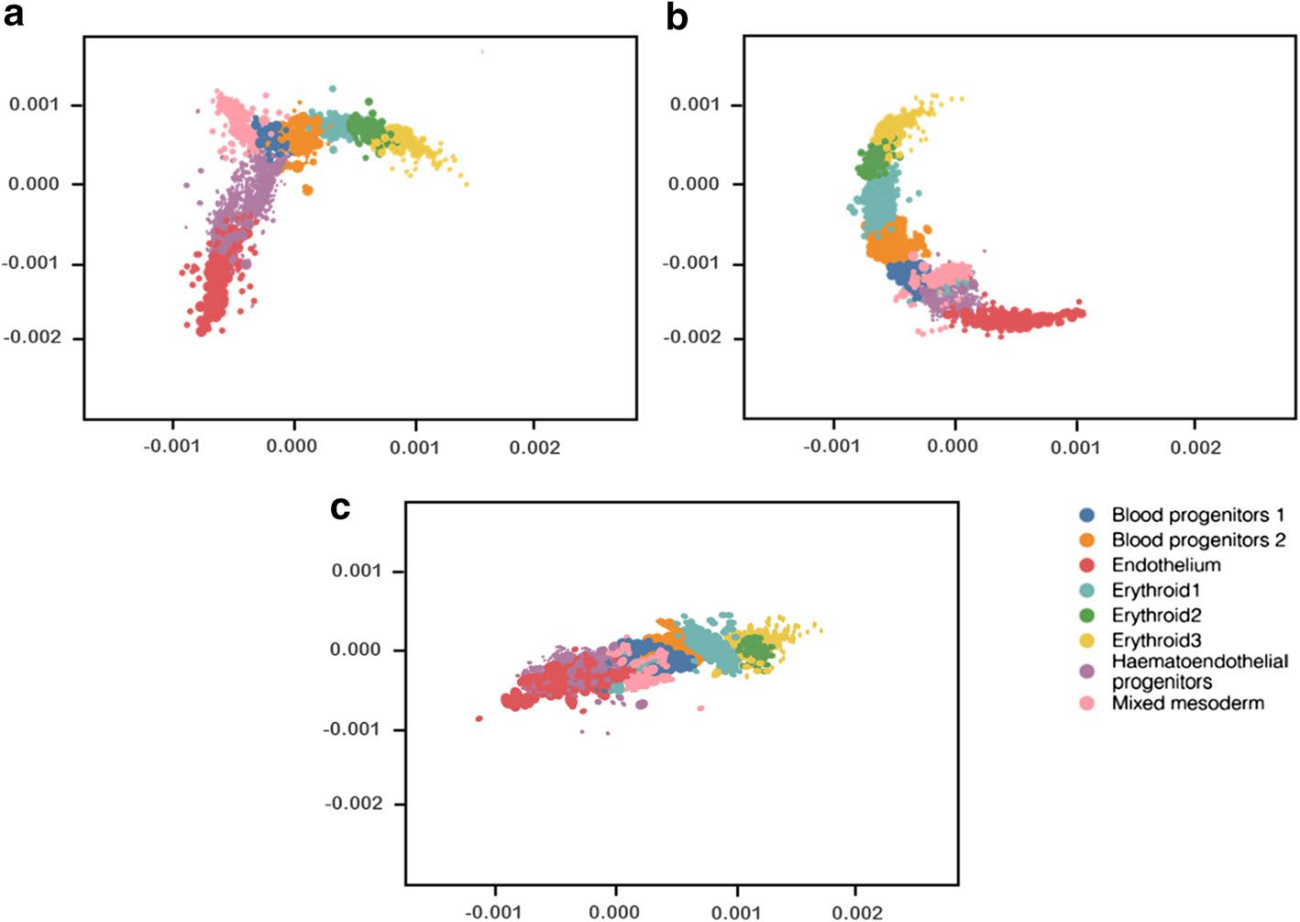
Time course developmental trajectories analysis. **a** Visualization for the trajectory analysis after Harmony, **b** visualization for the trajectory analysis after SSBER, **c** visualization for the trajectory analysis after LIGER

To explore the integrating performance of SSBER in analysis of time course developmental trajectories, we implemented the same experiment as Harmony [12]. The datasets include eight times points of mouse hematopoiesis, from E6.75 to E8.5 and mixed gastrulation. After data integration, we used the DDRTree method in the monocle package [28] to perform trajectory analysis, shown in Fig. 12. Both SSBER and Harmony recovered a branching trajectory structure that correctly captures the progression from common mesoderm and hematoendothelial progenitor populations to differentiated endothelial and erythroid populations. And SSBER also preserved the separation between the two blood progenitor populations and among the three erythroid populations. LIGER failed to present a clear branching trajectory structure and separate some distinct populations. MultiCCA failed to converge to an answer, as some of the samples contained too few cells, causing the Seurat optimization step to fail to converge.

### Robustness analysis

**Fig. 13:**
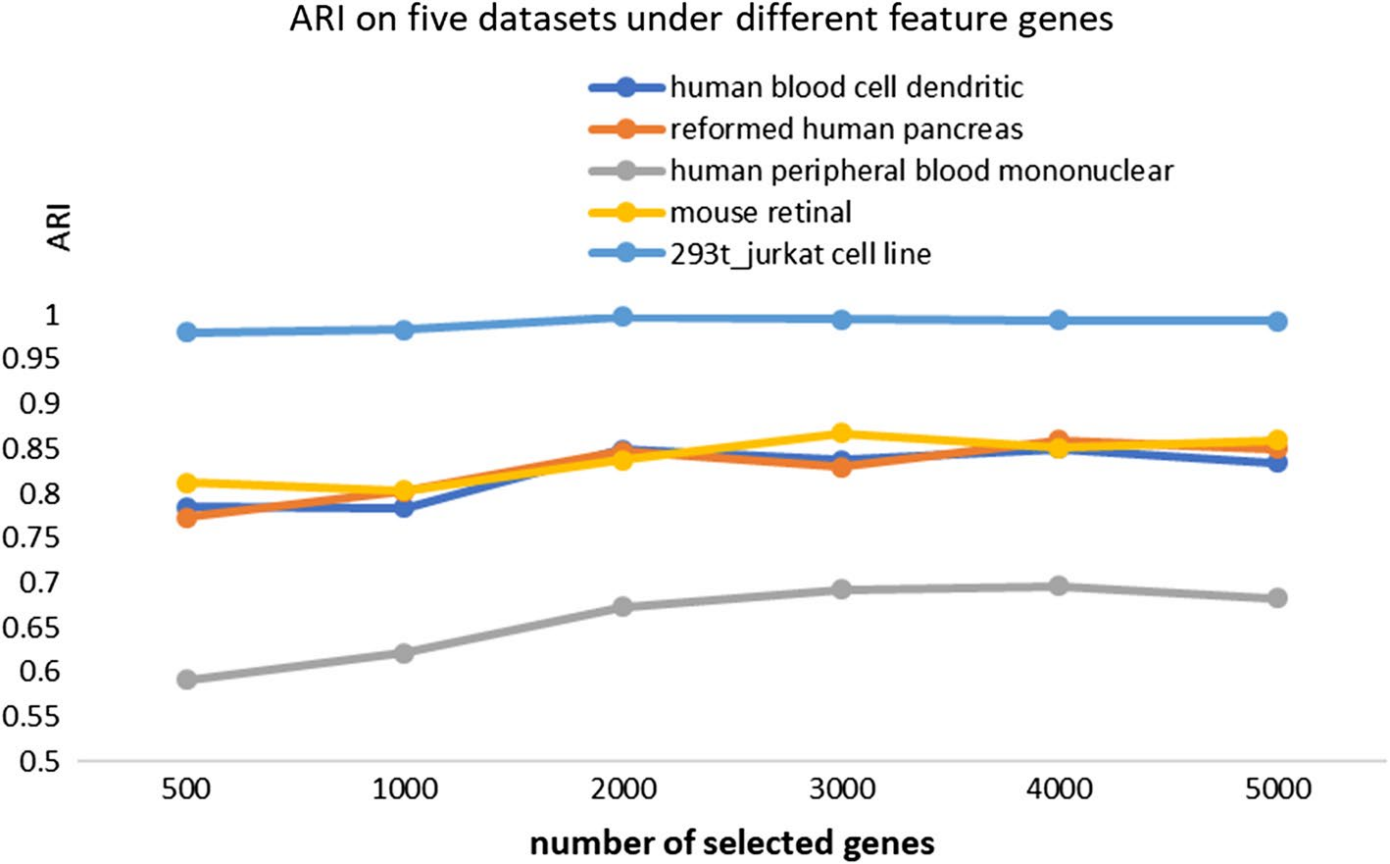
ARI scores under different numbers of genes

Determining some genes for scRNA-seq data analysis is to avoid curse of dimensionality. Usually, genes are selected based on the variance of expression abundance. In this section, we checked the robustness of SSBER for the number of selected genes. The results are shown in Fig. 13 in terms of ARI on the above five datasets. The number of genes with most variances are set as 500, 1000, 2000, 3000, 4000 and 5000 respectively. It can be seen that SSBER has good robustness since the ARI scores basically keep stable. The suggestion of the number of genes seems to be 3000 to 5000.

## Discussion

SSBER depends on a supervised classifier to label cell types with high precision. By now, SciBet is one of the best classifiers. It is easy for SSBER to transfer to other classifiers. At present, classifiers of more than 100 common cell type of humans and mice have been provided [29]. If some new tissues or new cell types are not covered in them, researchers could try to find the relevant labeled datasets to train corresponding classifiers. Although the current public datasets cannot support the needs of all human and mouse cell types, the human cell atlas and other animal cell atlases become more and more complete, the labeled datasets and corresponding classifiers will become more abundant. In the worst case, valid cell population labels cannot be provided, anchors could be identified without constraint of common cell type, SSBER degenerates to Seurat.

## Conclusions

In this paper, a method named SSBER to remove batch effect of scRNA-seq data is presented. SSBER considers the partial shared cell types predicted by a cell annotation algorithm and detects mutual neighbor cell pairs among the shared cell types, which improves the accuracy of anchors. Besides, batch effects are calculated for each cell type, unlike Seurat, Scanorama and BBKNN with global treatment. Therefore, when batches are highly heterogeneous, especially when the cell type structure among batches or distribution of cell population varies considerably, or some similar cell types exist across batches, SSBER outperforms other algorithms about integrating scRNA-seq data.

## Methods

To remove batch effect, it is ideal that some cells are sequenced in each batch which work as control to calibrate batch effects. Those same cells across batches act as anchors. Actually, cells of a same cell type are the realistic alternative of anchors. Usually, anchors are identified from pairs of cells, in which (1) two cells ($j_{1}$,$j_{2}$) come from two batches (B_1_, B_2_) and (2) $j_{1}$ is one of the k cells in batch B_1_ with the smallest distances to $j_{2}$, and vice versa $j_{2}$ is one of the k cells in batch B_2_ with the smallest distances to $j_{1}$. The differences between gene abundance of ($j_{1}$,$j_{2}$) represent batch effects.

Traditional data integration methods based on the anchor idea, such as MNN, Seurat, and BBKNN, must follow three assumptions [7]:

1. There is at least one cell population that is present in both batches (i.e., in the reference and the new batch to be merged with it).
2. The batch effects are almost orthogonal to the biological subspace.
3. Variation in the batch effects across cells is much smaller than the variation in the biological effects between different cell types.

**Fig. 14:**
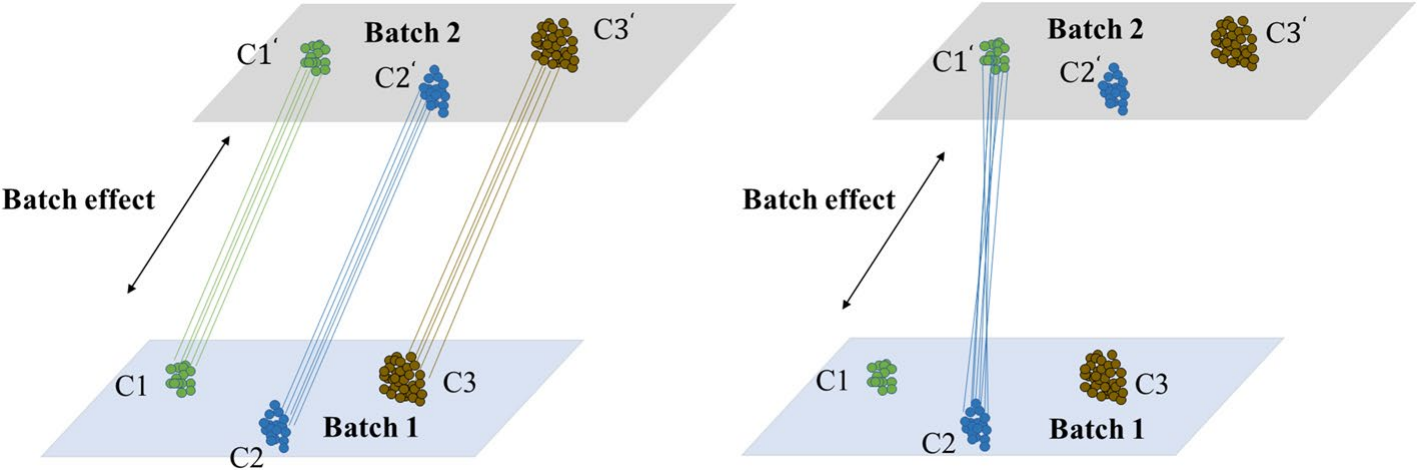
Detecting mutual nearest neighbors between two batches in non-orthogonal scenario

In fact, the assumptions might not hold up in real data, particularly given that different batches may easily differ in many aspects, including samples used, single cell capture method, or library preparation approach. If true biological variations are not orthogonal to batch effects, or differences from batch effect are not smaller than its from biological variations, traditional methods will meet a big challenge. Anchor pairs detected by KNN method may be cells from different cell types, misleading the batch-effect correction. For example, under the scenario depicted in Fig. 14, MNN leads to cluster 1 (C1) and cluster 2 (C2) mis-corrected due to mismatching single cells in the two clusters/cell-types across batches [6].

**Fig. 15:**
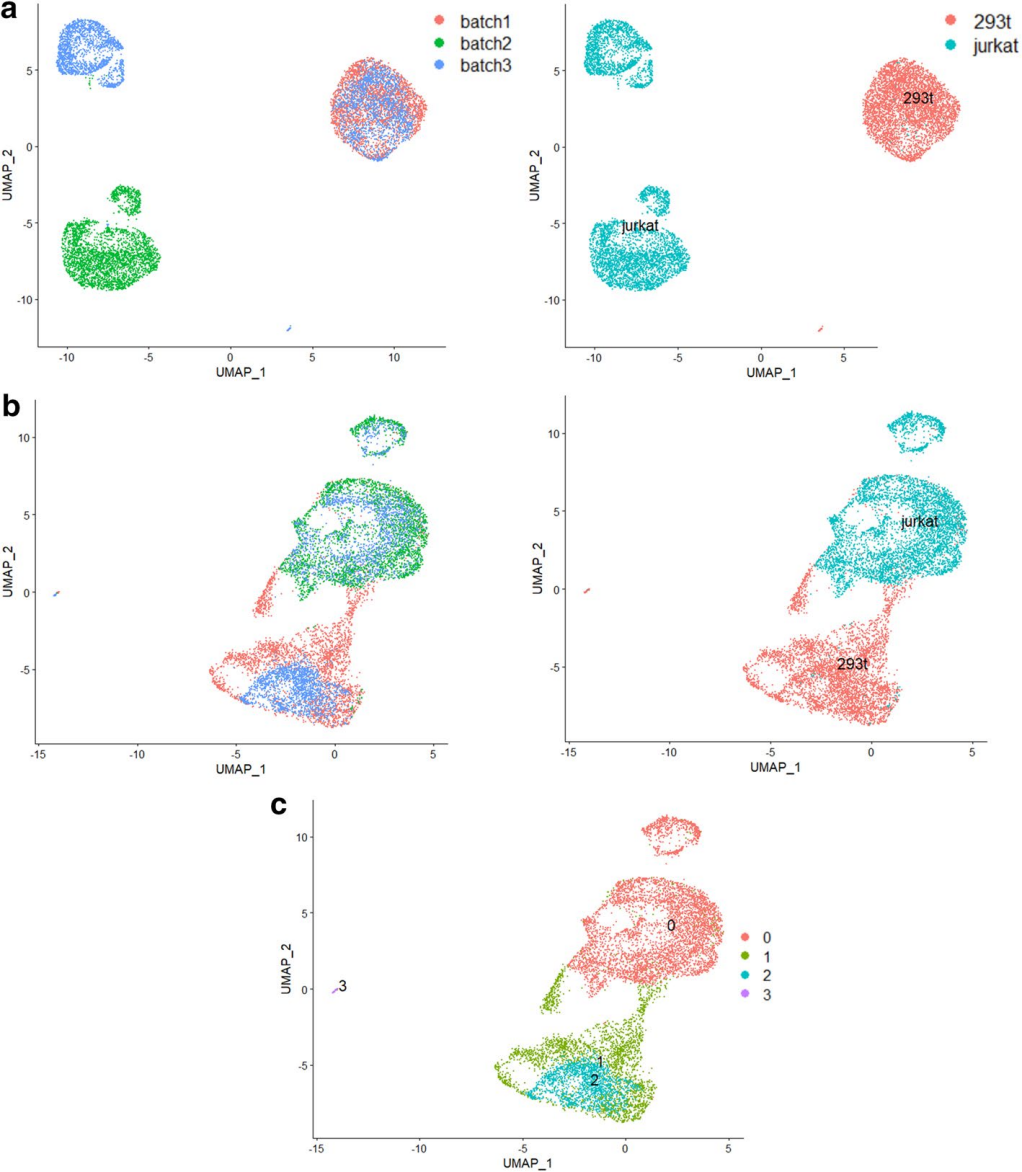
An example to illustrate the impact of anchor pairs. **a** Visualization for the cell line datasets, **b** UMAP analysis on the integrated data after Seurat, **c** clustering on the integrated data after Seurat

What’s more, we collected the cell line dataset [16] as an example to illustrate the impact of anchor pairs. The overall data visualization is shown in Fig. 15a. The datasets contain only two cell types, with two out of the three batches containing only one cell type that is also only shared with the third batch. Cell types, jurkat and 293t, appear separately in batch 1 and batch 2. After integration, Seurat or MNN produces four batch-mixed clusters, but with two cell types mixing (Fig. 15b, c), and the ARI (Adjusted rand index, one indication of clustering accuracy) only reaches 0.63, which seriously damages structure of the raw data. Through further analysis of the anchor pairs identified in Seurat, we find that about 63.13% of 8012 anchor pairs are not from the same cell type, that is, a large number of wrong anchor pairs seriously affect the final data integration performance.

To address those issues, here we present SSBER, a supervised method utilizing biological prior knowledge combined with anchor-based approach. The key idea of SSBER is to improve accuracy of detected anchors, two cells of which should come from a same cell type, consequently to improve performance of integration. SSBER first employs a supervised classifier to group cells with high confidence, then identifies anchors in shared cell type, which breaks the constraints that batch effects are orthogonal to the biological subspace and differences from batch effects are much smaller than its from biological variations.

The framework of SSBER is shown in Fig. 1, that includes four parts: (1) data preprocessing, Single-cell gene expression data from different batches are considered as input data and preprocessed according to the standardized process in Seurat software package (Fig. 1a). (2) Identification of shared cell types, SSBER utilizes SciBet (a single cell annotation tool) to annotate cell types (Fig. 1b), then identifies shared cell types. (3) Anchor detection, anchor pairs are detected in shared cell types (Fig. 1c). (4) Data integration, correction vectors for each cell are computed from anchor pairs (Fig. 1d, e).

### Data preprocessing

SSBER normalizes each cell using natural logarithmic transformation method with a factor of 10,000. Next, it uses z-score transformation to standardize the expression value of each gene. In order to avoid curse of dimension, top genes in variance are selected.

### Identification of shared cell types

After making a comparison of SciBet [29] with ScMap [30], Garnett [31], CellAsign [32] and so on, SSBER uses SciBet [29] to annotate cell type in each batch, consequently some shared cell types could be identified within labelled cells with high confidence. SciBet is a supervised model that predicts cell type for query data [29]. In order to ensure the annotation accuracy, the probability threshold of a cell type given by SciBet is set to 0.8, otherwise, cell type is assigned as unknown.

### Anchor identification

First, the raw data is mapped to a shared low-dimensional space through CCA (canonical correlation analysis). The typical correlation vector calculated by CCA can capture the shared signals between batches. Then KNN algorithm is employed to detect mutual nearest neighbors within a shared cell type in both original data space and low dimensional space. Those mutual nearest pairs in both spaces are identified as anchors [9].

### Data integration

SSBER calculates correction vector for each cell in combination with Gaussian kernel weights. More importantly, correction vector for a cell with a shared cell type is computed only from anchors within the same cell type, it could ensure distinguishing local batch effect on each cell type. If cell type is unknown, near anchors without cell type constraint are used to compute correction vector for a cell.

Since SSBER detects anchors only in shared cell types, it not only improve the accuracy of anchors which actually in biological motivation should come from a same cell type, but also in the case of multi-batch data integration, the final integration result will not be affected by the order of batch integration.

## Data Availability

SSBER is available and open source at (https://github.com/zy456/SSBER), the datasets we used are listed in the references and are available.

## Abbreviations

ASW: Average silhouette width
ARI: Adjusted rand index
CCA: Canonical correlation analysis
HVG: Highly variable gene
KBET: K-nearest-neighbor batch estimation
KNN: K-nearest neighbor
LISI: Local inverse Simpon’s index
scRNA-seq: Single-cell RNA sequencing
SVD: Singular value decomposition
t-SNE: T-distributed stochastic neighbor embedding
UMAP: Uniform manifold approximation and projection
